# Validation of Reference Genes for the Relative Quantification of Gene Expression in Human Epicardial Adipose Tissue

**DOI:** 10.1371/journal.pone.0032265

**Published:** 2012-04-12

**Authors:** Kanta Chechi, Yves Gelinas, Patrick Mathieu, Yves Deshaies, Denis Richard

**Affiliations:** Centre de Recherche de l'Institut Universitaire de Cardiologie et de Pneumologie de Québec, Université Laval, Québec, Canada; Naval Research Laboratory, United States Of America

## Abstract

**Background:**

Relative quantification is a commonly used method for assessing gene expression, however its accuracy and reliability is dependent upon the choice of an optimal endogenous control gene, and such choice cannot be made a priori. There is limited information available on suitable reference genes to be used for studies involving human epicardial adipose tissue. The objective of the current study was to evaluate and identify optimal reference genes for use in the relative quantification of gene expression in human epicardial fat depots of lean, overweight and obese subjects.

**Methodology/Principal Findings:**

Some of the commonly used reference genes including *18S*, *ACTB*, *RPL27*, *HPRT*, *CYCA*, *GAPDH*, *RPLPO*, *POLR2A* and *B2M* were quantified using real-time PCR analysis. The expression stability of these genes was evaluated using Genorm, Normfinder and Bestkeeper algorithms. In addition, the effect of sample size on the validation process was studied by randomly categorizing subjects in two cohorts of n = 2 and n = 33.

**Conclusions/Significance:**

*CYCA*, *GAPDH* and *RPL27* were identified as the most stable genes common to all three algorithms and both sample sizes. Their use as reference gene pairs might contribute to the enhanced robustness of relative quantification in the studies involving the human epicardial adipose tissue.

## Introduction

Human epicardial adipose tissue (EAT) is a visceral fat depot that has gained significant attention in the recent times. Numerous studies have reported significant positive correlations between EAT mass and coronary artery disease (CAD) in humans [1], [2], [3]. In addition, significant correlation is reported between visceral obesity and EAT mass [4], [5]. The current paradigm thus remains that increased EAT mass due to obesity increases the risk of developing CAD. However, the underlying mechanisms explaining this association remain unknown. EAT is a metabolically active depot capable of secreting various adipokines and cytokines. Moreover, it is located between myocardium and the inner layer of visceral pericardium, thereby sharing close proximity and a common blood supply with the underlying myocardium [6]. It is likely that EAT affects the cardiac-function and -metabolism in a paracrine manner. A number of recent studies have, thus, investigated the association between EAT expression of various adipokines, cytokines, oxidative stress- and inflammatory- markers with CAD [7], [8], [9], [10]. For these studies, relative quantification of gene expression remains the method of choice. In addition, our benign understanding of human EAT function would largely depend upon future studies assessing gene expression in this fat depot.

Relative quantification is an easy, quick and effective way of assessing gene expression, however its level of accuracy is dependent upon various experimental steps including handling of tissues, RNA extraction, storage of isolated RNA, efficiency of reverse transcription and amplification [11], [12]. Thus, it is a common practice to normalize the data against an endogenous reference gene or housekeeping gene (HKG) in order to correct for the potential experimental inaccuracies [13]. An ideal internal reference gene or HKG would be universally valid exhibiting stable expression across most sample types and experimental conditions, such that any differences in its expression could reflect upon the experimental variation leading to data correction. However, the literature suggests that no such gene exists, infact, the expression of the most commonly used HKGs can vary based on the experimental conditions and chosen set up [14], [15], [16]. The impact of using an unstable HKG can lead to erroneous results as demonstrated previously by Dhehda et al. and others [17], [18], [19]. Thus, it is crucial to identify and validate the HKGs prior to their use for normalization during specific experimental set ups.

To date, none of the studies dealing with human EAT has reported on the evaluation of HKGs prior to their use. Considering that differences in the expression of HKGs have been reported between omental and subcutaneous tissues [20], it becomes essential to validate the HKG to be used for the studies involving human EAT since various regional fat depots differ in their gene expression. In the current study, we have compared the expression of 9 commonly used HKGs in the EAT of lean, overweight and obese patients undergoing coronary artery bypass grafting (CABG). We employed the commonly used approaches of Genorm, Normfinder and Bestkeeper to identify the most stable HKGs. In addition, we randomly categorized our subjects in two cohorts of n = 12 and n = 33 in order to assess the impact of sample size on the validation approaches. We report that *CYCA*, *GAPDH* and *RPL27* are among the most stably expressed HKGs common to all 3 algorithms and both sample sizes in human EAT.

## Results

All the subjects (n = 33) included in the study underwent CABG. All of them were dyslipidemic, 54.5% had hypertension, 30.3% had diabetes, 24.2% had metabolic syndrome, 24.2% had peripheral vascular disease (PVD) and 30.3% smoked. All of the subjects were kept on statins and anticoagulants, 78.8% on beta-blockers, 51.5% on angiotensin converting enzyme-inhibitors, 9.0% on angiotensin-receptor blockers and 24.2% on oral hypoglycemic medication. Based on their body mass index (BMI), the subjects were divided into three categories of lean, overweight and obese. BMI and waist circumference of the obese group was significantly higher than the lean and overweight groups (*P*≤0.05) (Table 1). However, other clinical parameters including systolic- and diastolic- blood pressure, mean arterial pressure, age, fasting plasma glucose, triglycerides, total-, LDL- and HDL-cholesterol were not different among various groups (Table 1).

**Table 1:** Clinical characteristics of the subjects in the cohort.

Clinical characteristics	Lean (n = 9)	Overweight (n = 18)	Obese (n = 8)
Body mass index (kg/m^2^)	23.8 ± 0.5^c^	26.6 ± 0.2^b^	32.5 ± 0.6^a^
Waist circumference (cm)	93.0 ±1.6^b^	100.0 ±1.6^b^	114.3 ± 2.7^a^
Age (years)	62 ± 3.8	60 ± 2.7	59.4 ± 3.5
SBP (mmHg)	131.8 ± 9.03	125.8 ± 3.2	125.1 ± 3.06
DBP (mmHg)	68 ± 2.9	72.3 ± 2.1	72.4 ± 2.0
MAP (mmHg)	89.3 ± 2.8	90.1 ± 2.4	90.0 ± 1.6
FPG (mM)	6.4 ± 0.8	6.3 ± 0.5	5.8 ± 0.33
Total-cholesterol (mM)	4.1 ± 0.36	3.8 ± 0.18	3.7 ± 0.4
LDL-cholesterol (mM)	2.3 ± 0.3	2.1 ± 0.16	1.8 ± 0.17
HDL-cholestreol (mM)	1.3 ± 0.2	1.1 ± 0.06	1.1 ± 0.09
Triglycerides (mM)	1.4 ± 0.16	1.5 ± 0.17	1.9 ± 0.20

Superscripts represent statistically significant differences (*P*≤0.05) determined using one-way ANOVA and *Tukey's* post-hoc analysis. SBP =  systolic blood pressure, DBP =  diastolic blood pressure, MAP =  mean arterial pressure, FPG =  fasting plasma glucose.

In order to determine the expression stability of selected HKGs across these patient groups, we begun with calculating their respective PCR amplification efficiencies as the first step. The cDNA from randomly chosen lean, overweight and obese subjects were pooled, serially diluted and amplified for the preparation of a standard curve. The slope of the standard curve was then used for calculating PCR amplification efficiency according to the expression: E = −1+10^(−1/slope)^. Table 2 lists the amplification efficiency for each of the candidate HKG that ranged from 90–100%. Next, Genorm, Normfinder and Bestkeeper algorithms were employed to establish the expression stability of candidate genes for the sample sizes of n = 12 and n = 33. Genorm algorithm operates on the assumption that the ratio of two ideal reference genes should be constant under different experimental conditions. In contrast, Normfinder algorithm uses a model-based approach for identifying the most stable genes based on least inter- and intra-group expression variations. Bestkeeper identifies the most stable genes based on the coefficient of correlation to the bestkeeper index, which is generated by the geometric mean of the Ct values of best candidate genes under study.

**Table 2:** Candidate reference genes with respective symbol, accession number, name, primer sequences and efficiency of amplification (E).

Gene Symbol (Accession Number)	Gene Name	Primer Sequence (5′-3′)	E (%)
*RPLPO* (NM_001002)	Ribosomal protein large P0	F: GGATTACACCTTCCCACTTGCT R: GCCACAAAGGCAGATGGATCA	92
*RPL27 (*NM_000988)	Ribosomal protein L27	F: GTGAAAGTGTATAACTACAATCACC R: TCAAACTTGACCTTGGCCT	91
*HPRT* (NM_000194)	Hypoxanthine phosphoribosyl-transferase 1	F: ACCCCACGAAGTGTTGGATA R: AAGCAGATGGCCACAGAACT	91
*B2M* (NM_004048)	Beta-2 microglobulin	F: GCTATCCAGCGTACTCCAAAG R: CACACGGCAGGCATACTC	99
*ACTB* (NM_001101)	Beta-actin	F: CATCCACGAAACTACCTTCAACTC R: GCAATGATCTTGATCTTCATTGTG	95
*18S* (NR_003286)	18S ribosomal RNA	F: CAGCCACCCGAGATTGAGCA R: TAGTAGCGACGGGCGGTGTG	99
*POLR2A* (NM_000937)	RNA polymerase 2A	F: CTTCACGGTGCTGGGCATT R: GTGCGGCTGCTTCCATAA	95
*CYCA/PPIA* (NM_021130)	Peptidylprolyl isomerase A	F: ATCCTAGAGGTGGCGGATTT R: CACTCAGGTCTGAGCCACAA	90
*GAPDH* (NM_002046)	Glycerladehyde 3-phosphate dehydrogenase	F*:* ATGTTCGTCATGGGTGTGAA R: GGTGCTAAGCAGTTGGTGGT	97

### Genorm Analysis

**Figure 1:**
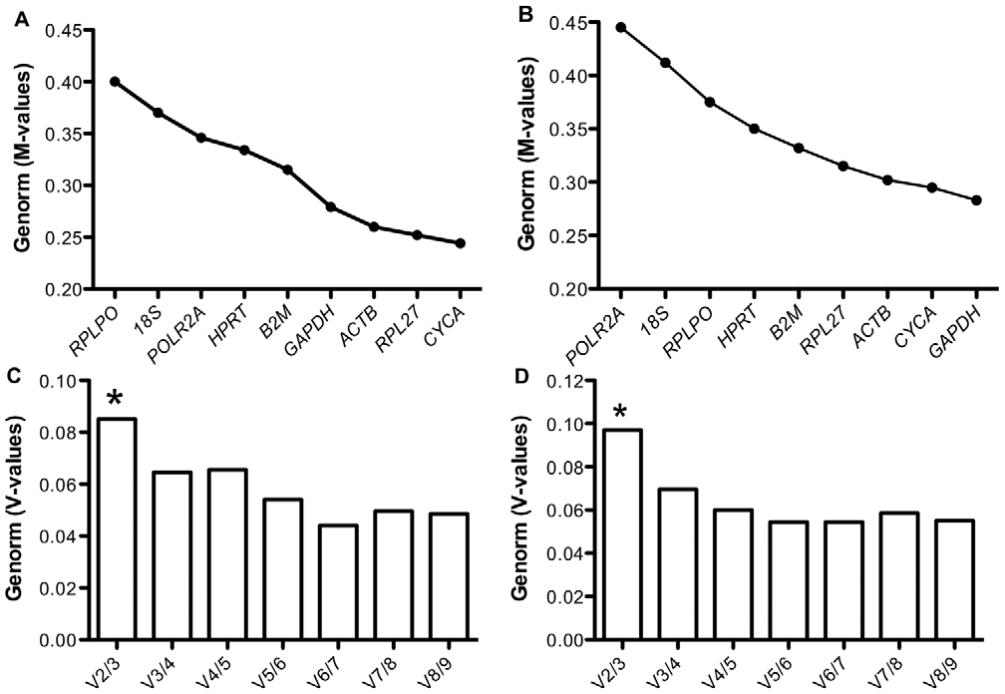
Validation of candidate genes using Genorm. Genorm M-values of the candidate genes for (A) n = 12 and (B) n = 33. Pairwise variation (V-values) of the candidate genes for (C) n = 12 and (D) n = 33. *represents the optimal number of reference genes required for the calculation of normalization factor.

Comparison of the raw non-normalized quantitative data using genorm revealed that most candidate HKGs exhibited expression stability (M) values below 0.5 for both sample sizes, suggesting that all of the 9 genes under study had stable expression. However, successive elimination of the least stable genes based on highest M-values led to the identification of *CYCA* and *RPL27* as the most stable genes for n = 12 (Figure 1A). In contrast, *GAPDH* and *CYCA* turned out to be the most stable genes when n = 33 was considered (Figure 1B), suggesting that Genorm analysis is sensitive to sample size. Indeed, the ranking of genes was different when either n = 12 or n = 33 was used for analysis (Figure 1A, Figure 1B), although for both sample sizes *RPL27*, *CYCA*, *ACTB* and *GAPDH* exhibited lowest M-values and hence the best expression stability.

In addition, Genorm calculated the number of optimal reference genes to be used for the derivation of a normalization factor (NF). With the pairwise variation calculated between two sequential NFs (NF_n_ and NF_n+1_), V_2/3_ exhibited the highest V-value below the cut-off value of 0.15 for both sample sizes (V = 0.084 for n = 12 and V = 0.097 for n = 33), indicating that use of 2 genes for normalization is necessary, whereas addition of a third gene is optional (Figure 1C, Figure 1D).

### Normfinder Analysis

**Figure 2:**
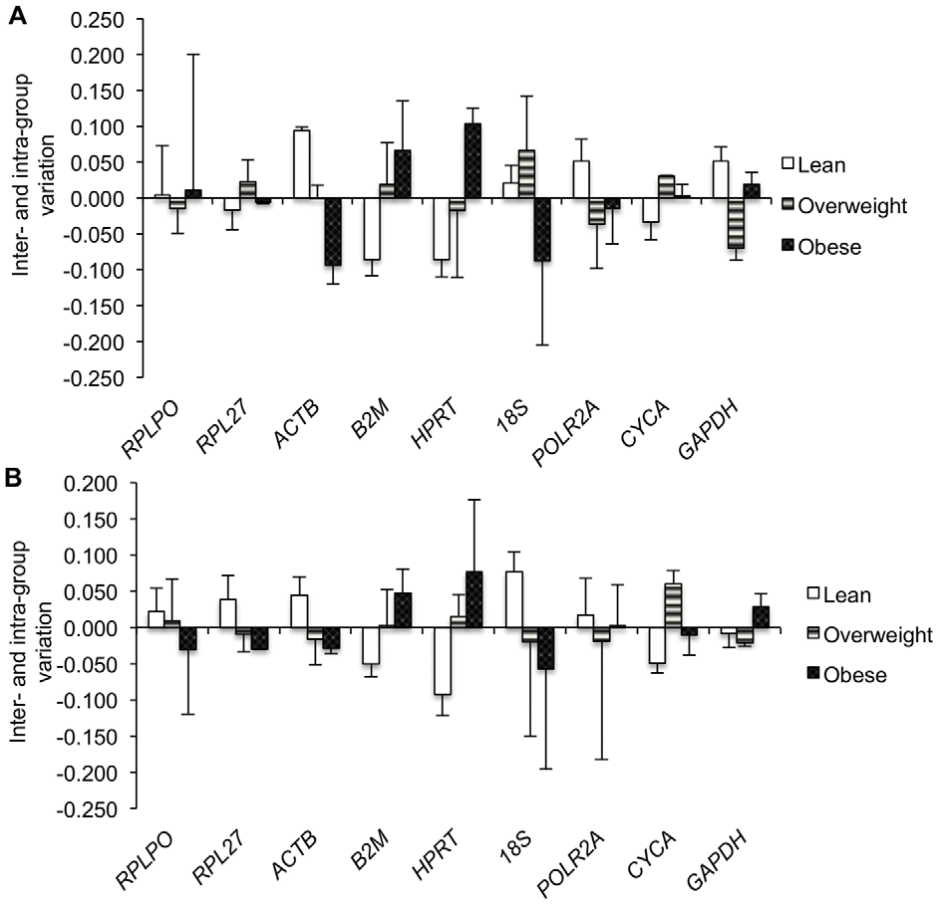
Validation of candidate genes using Normfinder algorithm. Inter- and intra-group variation of each candidate gene for (A) n = 12 and (B) n = 33. Columns represent the inter-group variation, whereas the error bars represent the intra-group variation for each candidate gene.

**Figure 3:**
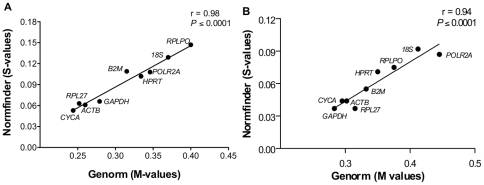
Determination of the stable genes common to both Genorm and Normfinder algorithms. Correlation analysis between M-values (Genorm) and S-values (Normfinder) representing the expression stability of each candidate gene for (A) n = 12 and (B) n = 33. The r-value signifies the coefficient of correlation. A *P≤*0.05 was considered to be significant.

In contrast to Genorm, Normfinder identified *CYCA* and *ACTB* as the genes with lowest S-values and hence the least variation index for n = 12, whereas *RPL27* and *GAPDH* had the lowest S-values for n = 33 (Table 3). However, comparison of the inter- and intra-group variation among lean, overweight and obese subjects revealed *RPL27*, *CYCA* and *GAPDH* to be the genes exhibiting lowest variation and hence highest stability for both sample sizes of n = 12 (Figure 2A) and n = 33 (Figure 2B). Since Genorm and Normfinder utilize different approaches for identifying stable genes, the observed differences in rankings between these two algorithms would be expected. However, considering that both assumptions are valid, a correlation analysis between M-values (Genorm) and S-values (Normfinder) for each candidate HKG was conducted such that most stable genes common to both algorithms could be identified. Indeed, *CYCA*, *RPL27*, *ACTB* and *GAPDH* clustered very closely on the correlation graph for both sample sizes of n = 12 (Figure 3A) and n = 33 (Figure 3B), thereby representing the most stable genes common to both Genorm and Normfinder.

**Table 3:** Gene stability (S) values calculated by Normfinder.

Candidate Genes	S-values (n = 12)	S-values (n = 33)
*CYCA*	0.053	0.044
*ACTB*	0.061	0.044
*RPL27*	0.063	0.037
*GAPDH*	0.066	0.037
*HPRT*	0.102	0.071
*POLR2A*	0.108	0.087
*B2M*	0.109	0.055
*18S*	0.129	0.092
*RPLPO*	0.147	0.075

### Bestkeeper Analysis

In order to qualify the observations common to Genorm and Normfinder, an independent approach used by the Bestkeeper algorithm was employed. Interestingly, for both sample sizes, *CYCA, GAPDH, RPL27 and ACTB* exhibited higher coefficient of correlation (r) to the bestkeeper index, lower coefficient of variance (CV) and standard deviation (SD), pointing towards their expression stability (Table 4). Although *POLR2A* and *B2M* exhibited higher r-values, they were not considered stable due to their higher CV- and SD-values (Table 4).

**Table 4:** Coefficient of correlation (r), coefficient of variation (CV) and standard deviation (SD) in the Ct values of each candidate gene calculated by the Bestkeeper algorithm for n = 12 and n = 33.

Candidate Genes	N = 12			N = 33		
	R	CV	SD	r	CV	SD
*POLR2A*	0.973	2.04	0.55	0.843	2.13	0.58
*CYCA*	0.966	0.97	0.22	0.845	1.40	0.32
*GAPDH*	0.916	1.72	0.40	0.931	1.67	0.39
*ACTB*	0.912	1.88	0.39	0.878	1.84	0.39
*RPL27*	0.897	1.58	0.35	0.853	1.42	0.32
*B2M*	0.858	2.23	0.44	0.902	2.26	0.45
*HPRT*	0.813	1.47	0.48	0.809	1.34	0.44
*18S*	0.701	1.96	0.33	0.486	1.84	0.32
*RPLPO*	0.687	1.97	0.41	0.742	1.82	0.38

#### Impact of clinical characteristics of the patients

Out of the 3 algorithms, only Normfinder is capable of determining the stability of candidate reference genes based on the sample type. Thus, we re-assessed the stability of the candidate genes using Normfinder after dividing the subjects into various categories based on their disease and medication status using n = 33. As shown in Table 5, *CYCA*, *GAPDH* and *RPL27* were invariably identified as the most stable genes in our cohort based on their smoking, PVD, diabetes, hypertension, MS and medication status. Indeed, these data support our conclusion that a combination of 2 genes out of *CYCA*, *GAPDH* and *RPL27* would represent the most stable reference genes across a variety of conditions for studies involving human EAT.

**Table 5:** Identification of most stable genes based on the disease and medication status of the subjects using Normfinder.

Condition	Best gene	S-values
**Disease status**		
*Smoking*	*CYCA*	0.029
*Peripheral vascular disease*	*GAPDH*	0.042
*Hypertension*	*GAPDH*	0.041
*Diabetes*	*RPL27*	0.048
*Metabolic syndrome* *	*RPL27*	0.032
**Medication status**		
*Beta-blockers*	*RPL27*	0.022
*Angiotensin converting enzyme- inhibitors*	*GAPDH*	0.025
*Angiotensin-receptor blockers*	*GAPDH*	0.023
*Oral hypoglycemics*	*RPL27*	0.054

* Information related to metabolic syndrome was available for n = 30.

## Discussion

Relative quantification of gene expression remains the method of choice, however its accuracy and reliability is critically dependent upon the choice of endogenous control or HKG [21]. While the use of an endogenous control is required for the correction of non-biological and experimental variation, a non-optimal endogenous control can either introduce pseudo-variation or mask the real biological variation leading to misinterpretation of data. Thus, it is essential to use an optimal reference gene for relative quantification. However, there is no “ideal reference gene” that could qualify the “one fits all” scenario, since most of the commonly used reference genes have been reported to be sensitive towards the experimental conditions and system under investigation [14], [15], [16]. Thus, it is advised to systematically validate the reference genes prior to their use for new experimental systems. In view of this, several algorithms designed to identify the most stable genes were developed [22]. We have used 3 of these very popular approaches, namely: Genorm, Normfinder and Bestkeeper to compare and validate a set of chosen HKGs, such that optimal reference gene/s could be identified to be used in the relative quantification studies involving human EAT. An ideal approach would be to perform a genome-wide survey of the human EAT, in order to identify the potentially stable HKGs, prior to implementing the validation process. However, due to the complexity and expensive nature of this approach, we restricted ourselves to the validation of a set of HKGs that are commonly used for studies involving EAT depots. In addition, considering the practicality of the validation process for each new experimental set up, we sought to identify the effect of sample size on the validation of HKGs. We randomly selected n = 4 from each of the lean, overweight and obese group of subjects from our cohort and created two sample sizes of n = 12 and n = 33 that were followed separately during the validation process.

Genorm algorithm uses a multiple pairwise comparison approach, where the expression stability (M) of a given gene is calculated as the mean standard deviation of the log-transformed expression ratios across samples relative to other reference genes remaining in the gene panel. This is followed by stepwise exclusion of individual gene with the highest M-value (*i.e.* the least stable gene) from the panel until reaching the last two genes with the smallest M-values (*i.e.* the most stable genes) [23]. Hellemens et al recommend using M≤0.5 for identifying most stable genes [24]. In addition, Vandesompele et al. recognized the error that is introduced when using a single HKG for normalization. Thus, in their landmark paper they introduced a mathematical approach to determine the optimal number of genes required for the calculation of a reliable NF [23]. Genorm uses this approach to calculate pairwise variation (V) between two sequential NFs *i.e.* NFn and NFn+1, until the variation drops below the recommended threshold of 0.15. Below this threshold, a larger v-value would indicate that the added gene has a significant effect and should be included for the calculation of a reliable NF. Using these parameters, Genorm identified that 2 most stable HKGs would be required for the calculation of a NF for both sample sizes. *CYCA* and *RPL27* were recognized as the most stable genes for a smaller sample size (n = 12), whereas *GAPDH* and *CYCA* were identified as the most stable genes for the larger sample size (n = 33). It is interesting to note that in each case, *CYCA*, *RPL27*, *GAPDH* and *ACTB* were identified as the top 4 stable genes. Ling et al. have previously reported altered expression stability/rankings of candidate genes (with Genorm analysis) in different sample subsets of Drosophila brains modeling aging related neurodegeneration, even when the samples had similar tissue composition [25]. Ling et al. thus concluded that expression stability of candidate HKGs is sample- and analysis-specific. Since Genorm computes its M-values based on expression ratio of candidate genes (multiple pair-wise comparisons), it is independent of variation in the amount of starting material as well as of the normal distribution of data. However, it does not correct for inter-group variation that is introduced when working with heterogeneous populations. As mentioned before, we begun with randomly selected (n = 4) subjects for each category of lean, overweight and obese subjects for n = 12 analysis. Whereas additional lean (n = 5), overweight (n = 12) and obese (n = 4) subjects were used for subsequent analysis of n = 33. It is likely that expression variation within each group of subjects could have led to different gene rankings for each sample size in our study.

The issue of intra- and inter-group variation and its impact on reference gene expression was addressed by Anderson et al [26]. They developed a model-based approach known as Normfinder to identify candidate reference genes with least inter- and intra- group variation. Thus, a stable reference gene according to Normfinder would have an inter-group variation close to zero with least intra-group variation. Interestingly, *CYCA*, *GAPDH* and *RPL27* were identified as the genes exhibiting least inter- and intra-group variation for both sample sizes. However, the gene rankings were different between Genorm and Normfinder. Considering the different approaches used by Genorm and Normfinder, it is expected that when two genes would exhibit higher expression variation across samples/groups they would be ranked lower with Normfinder even if their expression ratios do not change, thereby receiving a better M-value with Genorm. Since both of these approaches use valid assumptions, we performed a correlation analysis to identify best HKGs that would be common to both algorithms. Once again, *CYCA*, *RPL27*, *ACTB* and *GAPDH* were identified as the best 4 genes clustering very close on the correlation graph.

In contrast to Genorm and Normfinder, Bestkeeper analysis chooses stable genes based on the low variation of expression within the samples of tissue under study [27]. Bestkeeper calculates the coefficient of correlation (r-values) between each candidate gene and the bestkeeper index, which represents the geometric mean of best candidate genes. Thus, a higher r-value would correspond to stable expression of the candidate gene in the chosen experimental set up. In addition, Bestkeeper calculates standard deviation (SD) and coefficient of variation (CV) among Ct values across samples that help to identify the stability of a candidate gene. It is advised that a gene with SD value <1, low CV and higher r value would have stable expression across the tested set of samples. All of the 9 tested genes exhibited SD values <1, qualifying them as stable genes. However, *CYCA, GAPDH, RPL27* and *ACTB* were considered as the most stable genes due to their higher r- and lower CV-values for both sample sizes. In addition, when the data was re-analyzed using the disease and medication status of patients in our cohort, *CYCA*, *GAPDH* and *RPL27* turned out to be the most stable genes out of a pool of 9 otherwise stable genes (as pointed by the Genorm M-values and Bestkeeper SD values).

In conclusion, *CYCA*, *GAPDH* and *RPL27* were identified as the most stable reference genes common to Genorm, Normfinder and Bestkeeper algorithms for studies involving human EAT, not only in context of obesity but also under a variety of other conditions. Indeed, a combination of 2 genes out of these 3 genes would contribute to enhanced robustness of relative quantification analysis thereby impacting our current and future understanding of the epicardial fat depot in humans.

## Methods

### Ethics Statement

The study was approved by the ethics committee of the Institut Universitaire de Cardiologie et de Pneumologie de Québec. A written informed consent was obtained from all participants.

### Samples

EAT corresponds to the adipose depot in direct contact with the heart located between the myocardium and the visceral pericardium. EAT samples were collected from 33 patients undergoing CABG at the Institut Universitaire de Cardiologie et de Pneumologie de Québec, QC, Canada. The patients were divided into lean (n = 9, BMI<25.0), overweight (n = 16; BMI>25.0–30.0) and obese (n = 8; BMI>30.0) based on the BMI criteria used by the World Health Organization. The samples were collected in liquid nitrogen during the CABG procedure and stored at −80^°^C until further analysis.

### Selection of Reference genes

Candidate reference genes commonly used for the data normalization in the studies involving epicardial and other adipose tissues were selected for validation. These genes included: *18S* [7], [8], [28], [29], *B2M* [30], [31], *CYCA* [32], [33], [34], *HPRT* [35], *GAPDH* [36], [37], [38], [39], *ACTB* [10], [40], [41], *RPLPO* [42], *RPL27* [43], [44] and *POLR2A* [45], [46], [47].

### RNA extraction, reverse transcription and Quantitative PCR

Total RNA was isolated from 100 mg of tissue using the RNeasy Lipid Tissue Mini Kit (QIAGEN, Mississauga, Ontario) according to manufacturer's instructions. Purity of total RNA was determined as 260/280 nm absorbance ratio with expected values between 1.8–2.0 using a Multiskan Spectrum (Thermo Scientific, Milford, MA, USA). In addition, RNA integrity of randomly selected samples (n = 12) was assessed using the Bio-Rad Experion (Bio-Rad Laboratories, ON, Canada). Five hundred ng of extracted total RNA was reverse transcribed using Expand Reverse Transcriptase (Roche Diagnostics, Montreal, QC, Canada) according to the manufacturer's instructions. The cDNA was diluted 1:20 in DNase-free water before using for quantification by real-time quantitative PCR (qPCR). The real-time PCR mixture was prepared using SYBR® Green JumpStart™ Taq ReadyMix™ (#S5193, Sigma Aldrich, USA) according to the manufacturer's instructions. The primers for qPCR were designed using AlleleID (PREMIER Biosoft International, USA) and synthesized commercially (Invitrogen, USA). All primers were confirmed using the NCBI Blast tool against all available mRNA sequences to ensure specificity. The sequence for each set of primers is given in Table 2. The qPCR was performed in a 384-well plate format using the ABI-7900 HT Fast Real-time system (Applied Biosystems, USA). At the end of each run, melting curve analysis was performed and a few representative samples were run on agarose gel to ensure the specificity of the amplification. All samples were amplified in duplicates from the same RNA preparation and the mean values were used for further analysis.

### Determination of reference gene expression stability

To assess the stability of candidate reference genes, 3 commonly used approaches Genorm, Normfinder and Bestkeeper algorithms were utilized. Genorm^plus^ was downloaded as part of the Qbase^plus^ software available from http://medgen.ugent.be/~jvdesomp/genorm/. Normfinder was downloaded and used as an excel applet from http://www.mdl.dk/publicationsnormfinder.htm. In addition, the Bestkeeper algorithm was downloaded from http://www.gene-quantification.de/bestkeeper.html and used as an excel macro according to the developers instructions.

### Statistical analysis

Clinical characteristics of patients were compared among various groups using one-way ANOVA followed by Tukey's *post-hoc* analysis. Differences exhibiting a *P*≤0.05 were considered significant. Pearson's correlation analysis was used to determine the association between gene rankings obtained by Genorm and Normfinder. All statistical analysis was performed using Graphpad prism 5.0 software, La Jolla, CA, USA.
